# Compliance with the first UK covid-19 lockdown and the compounding effects of weather

**DOI:** 10.1038/s41598-022-07857-2

**Published:** 2022-03-09

**Authors:** Michael Ganslmeier, Jonathan Van Parys, Tim Vlandas

**Affiliations:** 1 Department of Social Policy and Intervention, Barnett House, 32-37 Wellington Square, Oxford, OX1 2ER UK; 2 YouGov, 50 Featherstone Street, London, EC1Y 8RT UK; 3 St Antony′s College, University of Oxford, 62 Woodstock Rd, Oxford, OX1 2ER UK

**Keywords:** Public health, Human behaviour

## Abstract

The effectiveness of containment measures has been shown to depend on both epidemiological and sociological mechanisms, most notably compliance with national lockdown rules. Yet, there has been growing discontent with social distancing rules during national lockdowns across several countries, particularly among certain demographic and socio-economic groups. Using a highly granular dataset on compliance of over 105,000 individuals between March and May 2020 in the United Kingdom (UK), we find that compliance with lockdown policies was initially high in the overall population during the earlier phase of the pandemic, but that compliance fell substantially over time, especially among specific segments of society. Warmer temperatures increased the non-compliance of individuals who are male, divorced, part-time employed, and/or parent of more than two children. Thus, while *epidemiologically* the virus spread was naturally more limited during the warmer period of 2020, *sociologically* the higher temperature led to lower individual-level compliance with public health measures. As long as new strains emerge, governments may therefore be required to complement vaccination campaigns with targeted and time limited restrictions. Since non-complying individuals at the beginning of the pandemic share certain characteristics with vaccination sceptics, understanding their compliance behaviour will remain essential for future policymaking.

## Introduction

In 2020 alone, the covid-19 pandemic cost over 1.8 million lives^1^ and led to more than 33 million layoffs worldwide^2^. To limit the spread of the virus, governments around the world resorted to imposing large-scale containment measures, most notably lockdowns on authorized economic activity, mobility and social interactions^3^. Several studies have demonstrated the crucial importance of isolating infected individuals and the necessity at the time to impose these restrictions on individual behavior to limit the initial spread of the disease^3–5^. Others have explored compliance (or intention to comply) with lockdown restrictions with an emphasis on effectiveness. For instance, it has been shown that compliance depends on partisanship^6,7^, gender^8^, news media^9^ and civic capital^10^.

One important but unanswered question concerns the role of weather and how it influenced the willingness of individuals to comply with public health and social distancing measures as the year 2020 progressed into warmer and dryer months. Previous research has shown that weather has an important impact on mobility and the whereabouts of a person^11–14^. However, these weather-based changes in mobility do not necessarily translate into a change in the rate of social encounters between individuals, implying a limited role of weather on rate of infection during the pandmic^11^. In contrast to conventional wisdom, recent evidence suggests no linkage between weather and mental health during lockdown^12^.

In this article, we contribute to this literature by testing whether and how climatological factors have shaped the willingness of individuals to comply with the initial government restrictions during the initial phase of the pandemic, most notably the social distancing rules and restrictions on individuals leaving their homes. Since lockdown measures are potentially very costly to people—both in economic and psychological terms—especially for certain individuals^15–17^, one could posit that the willingness of these groups to follow governmental guidelines declined as the opportunity costs of compliance increased with the increase in temperature as the year progressed. Thus, even if good weather may limit the virus spread in epidemiological terms and it does not significantly change the rate of social encounters as Wu et al. found, increasing non-compliance among certain citizens may have impaired the effectiveness of containment measures during periods of higher temperature.

The UK is an ideal case study for investigating compliance because it experienced a particularly severe early pandemic period coupled with a long-lasting and restrictive first national lockdown including social distancing regulation, school closure, restrictions on leisure activities, and a call to work from home from mid-March 2020 onwards^18^. To analyze whether and how climatological conditions changed the association between demographic as well as socio-economic factors and non-compliance during the first national lockdown, we rely on a large survey by YouGov that recorded over 105,000 individual responses about (declared) compliance behavior nested in 147 sub-national administrative units in the UK between 23rd of March and 18th of May 2020. This dataset represents one of the largest individual level surveys recording lockdown compliance behavior over time in a single country and its highly geographically granular structure allows us to match individual responses to weather conditions at a particular time *and* in a specific place.

Our results show that compliance with lockdown policies was initially high in the overall population during the earlier phase of the pandemic, but that compliance fell substantially over time and this effect was especially pronounced among specific segments of society. Warmer temperatures increased the non-compliance of individuals who are male, divorced, part-time employed, and/or parent of more than two children. Thus, the UK government now faces a challenge: the vaccine sceptics share many characteristics with the non-compliers. This may limit the effectiveness of policies to impose limited restrictions on non-vaccinated populations as well as policies to enforce compliance with vaccination programs. Although the UK has currently achieved high vaccination rates and vaccines appear effective against severe illness, the possibility of more vaccine resistant variants in the future may require public health measures and vaccination campaigns to be re-introduced in the years to come. As a result, finding strategies to ensure compliance and vaccination across reluctant social groups will be crucial to effectively respond to similar public health crises in the future.

## Results

### Non-compliance steadily increased over time during initial lockdown

**Figure 1:**
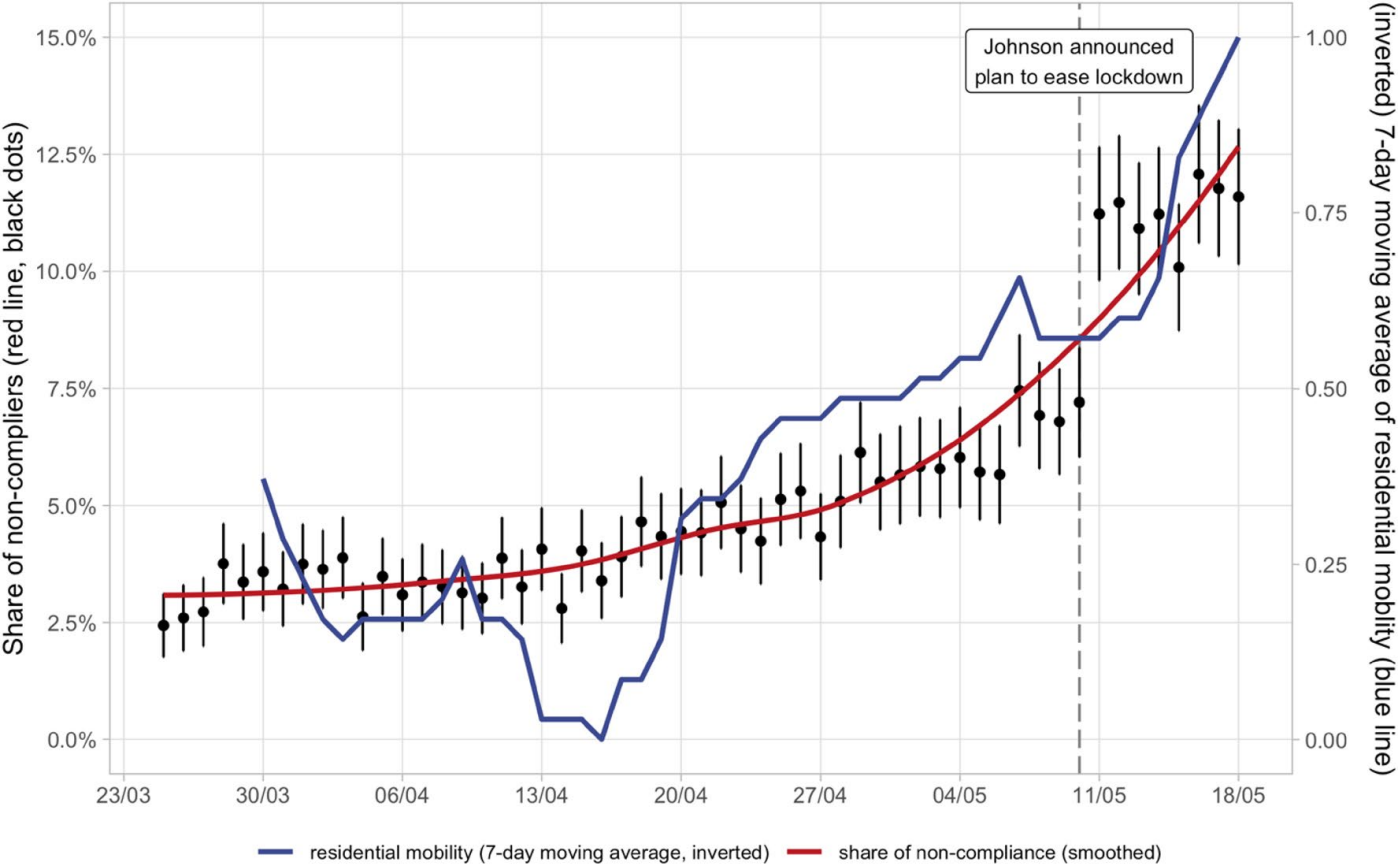
The share of non-compliers steadily increasing from end of March until mid of May. On the 10th of May 2020, Prime Minister Johnson announced the government’s plan of exiting lockdown, which led to a significant jump of the share of non-compliers. The plot shows the average non-compliance shares for each day (black dots) with the corresponding 95% confidence intervals represented by the vertical lines (sample weights are applied). The red line is a smoothing function based on non-compliance averages from previous days. The blue line represents the (inverted and rescaled to a 0–1 range) 7-day-moving-average of the residential mobility indicator provided by Google. The minimum in the mobility index in mid-April 2020 referred to the time when Prime Minister Johnson was admitted to the St Thomas’ hospital in London. N = 105,512.

Figure 1 plots the average share of non-compliance responses for each day during the time period covered in the dataset with 95% confidence intervals (represented by the vertical lines). It also displays a smoothed function of the weighted conditional means based on previous values of the daily average response (blue line). Overall, an upward trend in non-compliance during the first lockdown is clearly visible with an accelerating rate when the plan for re-opening was announced on 10th of May 2020. This upward trend of non-compliance was mirrored by the rising average temperature in the UK in the same period (SI. Fig. 1A). Moreover, there was also a notable spike in non-compliance responses observable when Prime Minister Johnson announced the government’s plans to ease lockdown on May 10, 2020. Although this is not the primary focus of the present article, we note that the communication strategies of containment policies appear to have had a significant impact on subsequent changes in the compliance responses.

One possible concern with this indicator is that it is based on self-declared compliance which could be subject to a social desirability bias. This in turn could lead to discrepancies between reported and actual behavior of respondents. To address this concern and test the reliability of our indicator, we carried out two separate checks. First, we cross-validated our self-reported individual level non-compliance measure with a real proxy of behavior. Figure 1 shows that the average daily compliance shares in the survey closely followed (correlation of 0.85) the 7-day moving average macro-level Google mobility residential indicator that does not depend on individual declarations but instead on actual behaviors (red line)^19^. There was a slight deviation in mid-April when Prime Minister Johnson was in hospital but interestingly during this period individuals appeared to be less mobile than they declared, which is not consistent with the presence of a social desirability bias.

**Figure 2:**
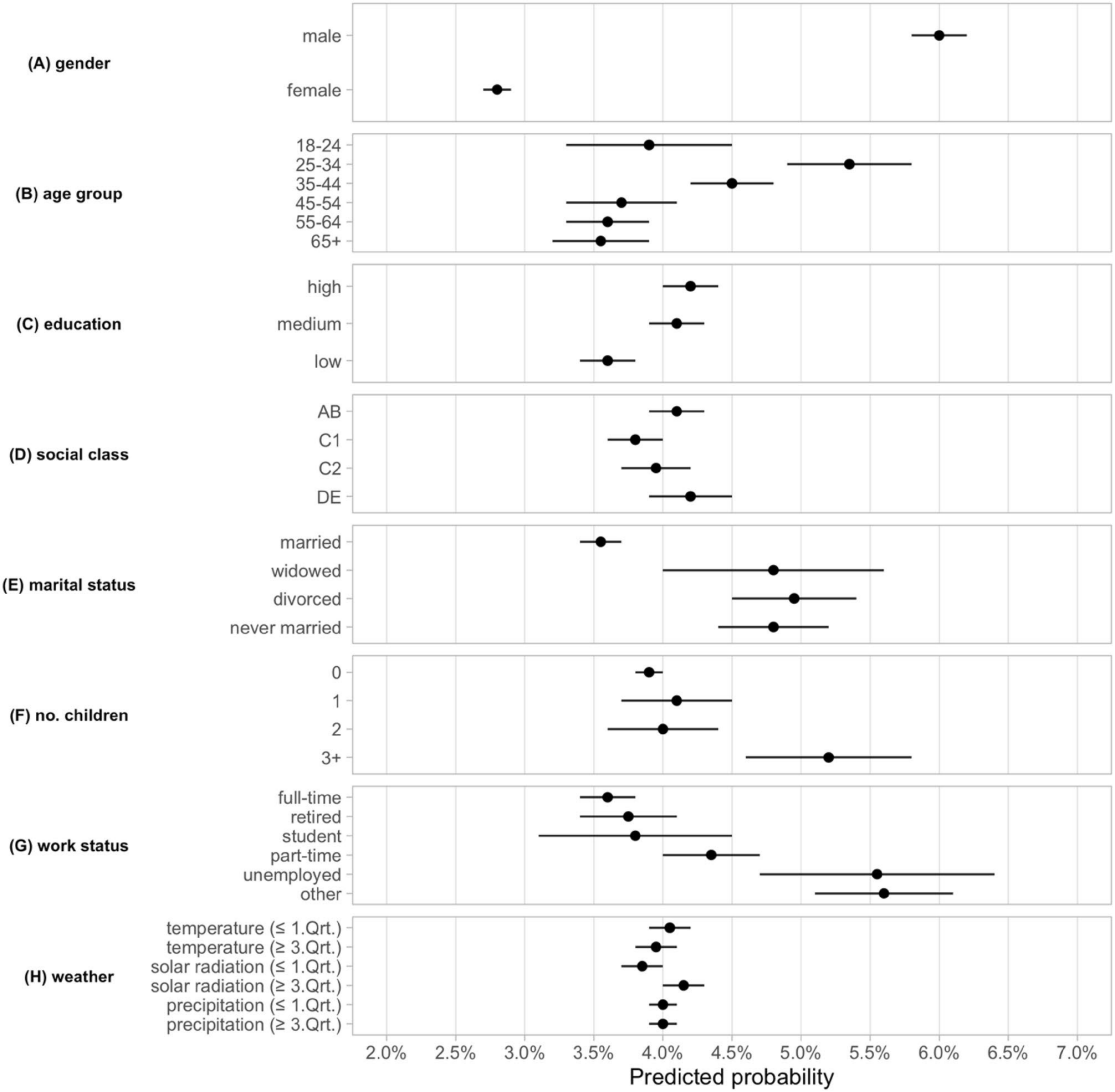
The predicted probabilities (in %) for non-compliance with lockdown policies were largest for male, young, unemployed, non-married ones or parents with more than two children. The plot shows the predicted probability of non-compliance when each individual level characteristic is equal to 1, while all other covariates are held constant at their mean values. The estimates are based on a logistic regression with date and region fixed effects included and robust standard errors clustered at the region level. The horizontal lines show the 95% confidence intervals. N = 105,512.

Second, since the google mobility indicator may on the other hand suffer from an ecological fallacy due to the higher level of aggregation, we carried out an additional check of our compliance measure. Specifically, we compared answers to the question about compliance with government policy with answers to questions about actual behavior of the respondents. As SI. Fig. 2 shows, respondents who reported that (A) they left home for a longer period, (B) they went to pursue fun activities, or (C) they did not focus extensively on self-isolation, all indicated higher shares of reported non-compliance than their respective peer groups. Thus, both micro-level survey questions and macro-level mobility data confirm that our compliance measure is a good proxy of actual compliance.

### Non-compliance strongly depended on socio-economic characteristics

Figure 2 plots the predicted probabilities (with 95% confidence intervals) of the individual-level characteristics and the weather indicators with all other control variables being held constant at their mean values (see SI. Table 2, SI. Table 3, SI. Table 4, and SI. Table 5 for robustness checks). Even though a vast majority of respondents declared that they followed the government’s lockdown guidelines (~ 95% among all survey participants), our results further suggest that there was large variation across individuals depending on their demographic as well as socio-economic characteristics which were strong predictors of their non-compliance. Five individual-level characteristics played a particularly important role in accounting for non-compliance.

First, the individual characteristic with the largest effect on non-compliance during the initial national lockdown was gender: female respondents had a predicted probability below 3% of non-compliance compared with 6% for male respondents. In other words, the likelihood of non-compliance was more than twice as large for male than for female respondents. This finding is in line with previous results showing that compliance was significantly higher among women than men^8^.

Second, younger individuals between 25 and 34 (and to a lesser extent, respondents between 35 and 44) had a significantly higher predicted probability of following governmental restrictions during the first lockdown, when compared to people above 45. These results are prima facie only partly consistent with evidence that mortality rates were higher for older individuals: while it is true that people between 45 and 54 had higher risks than people who were younger, we observe limited difference in non-compliance between individuals above this age. Thus, the effect of mortality risks on individual incentives to follow government policies only appeared for economically active individuals. Surprisingly, very young adults between 18 and 24 did not significantly differ from people aged above 45, although note the larger confidence interval. This higher non-compliance among some younger cohorts during the initial lockdown provides an interesting parallel to more recent findings about younger individuals’ greater reluctance to vaccinate^20^.

Third, labor market status had a sizeable influence on non-compliance. The unemployed had a 5.6% predicted probability of non-compliance compared to 3.6% for individuals with a full-time contract. Similarly, respondents with more precarious contracts (part-time and others) tended to have higher predicted probabilities of non-compliance than workers in more standard employment. There were no statistically significant differences between students, retired people and full-time employed people. Thus, precariousness rather than economic activity *per se* appeared to have shaped incentives for compliance.

Fourth, marital status and family size also displayed substantial differences. Marriage increased the predicted probability of compliance, whereas having more than two children had the opposite effect. Interestingly, being ‘not married at all’, ‘widowed’, or ‘divorced’ exhibited similar predicted probabilities of non-compliance. These findings are in line with previous results showing that loneliness and intensifying family conflicts in larger households generated larger psychological costs on certain groups of society during the first national lockdown^16^.

Fifth, the effects of social class and education on non-compliance with first national lockdown were more mixed. Class appeared to have no statistically significant effect, which is surprising given the potential differences in incomes across classes as well as the distinct material costs of complying with injunctions to work from home between different social classes. However, lower education was significantly associated with a lower predicted probability of not following government rules. This result runs against much conventional wisdom at the time and was not driven by multi-collinearity: correlation between education and social class is not high and including only one factor at a time does not change our findings (see SI. Table 4).

### Warmer temperatures increased non-compliance

Even though the seasonality of the pandemic and its dependence on climatology has been the focus of much epidemiology research^21,22^, weather has mostly been overlooked in compliance studies, despite having potentially relevant effects on social behavior. Exploiting highly granular data^23^, we were able to match each respondent to the exact time of interview and location. This is important because weather conditions varied widely across regions and over time during the survey period (see SI. Fig. 1).

**Figure 3:**
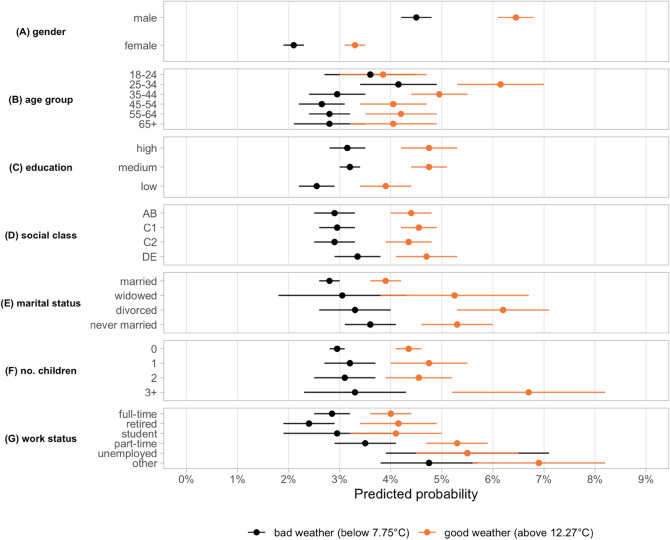
Warmer temperature had a large effect on non-compliance behavior with certain demographic and socio-economic characteristics. The panels show the predicted probability of non-compliance when a given individual level characteristic is equal to 1 while all other covariates are held constant at their mean values. The black (orange) dots are estimates based on observations under bad (good) weather conditions, where good and bad weather are defined by the top and bottom quartiles (12.27 °C and 7.75 °C , respectively) of temperature. The estimates are based on a logistic regression with date and region fixed effects included and robust standard errors clustered at the region level. The horizontal lines show the 95% confidence intervals. N = 105,512.

Overall, as the bottom panel of Fig. 2 illustrates, non-compliance was not generally associated with weather after controlling for individual-level, regional and time-varying factors. However, analyzing how compliance of individuals with specific characteristics was affected by weather conditions reveals that this null overall effect hides substantial amount of individual heterogeneity. When splitting the sample along key weather indicators, we find that certain socioeconomic groups displayed a strong reaction to changing weather conditions (Fig. 3—see also SI. Fig. 5, SI. Fig. 6), while others remained largely unaffected. The heterogeneous effects of weather are substantial for temperature, moderate for solar radiation, and statistically insignificant for precipitation.

Moreover, the largest amplification of the predicted probabilities under warm temperature conditions could be observed for four individual level characteristics: being male, being divorced, being part-time employed, and having more than two children. For instance, while the probability of non-compliance for female respondents rose from 2.1% under cold temperature to 3.2% under warm temperature, for male respondents the probability increased from 4.5% to 6.5% between cold and warm periods. Divorced individuals as well as parents of more than 2 children showed an even greater sensitivity to temperature changes with non-compliance probabilities in warmer environments being more than twice as large compared to colder periods.

Overall, our results are in line with an ‘opportunity cost’ logic: when temperature levels were high, compliance became a more costly choice—especially for the young, single individuals and large families—as the missed (social) opportunities were greater on warm days compared to cold periods of the year. Thus, while a broad range of epidemiological studies documented a seasonality in the spread of the virus with lower incidence in times of good weather^21,22^, our findings reveal the potential presence of an opposite social mechanism operating through changes in social behavior. This greater non-compliance might in turn have facilitated the transmission of the virus on warm and sunny days in the first half of 2020.

## Discussion

Although a large majority reported that they followed the government guidelines (~ 95%), the share of non-compliers increased steadily in the early period of the pandemic. Importantly, non-complying citizens shared common demographic and socio-economic characteristics at the individual level. Our results show that being male, aged between mid-20 s/early-30 s, unemployed, non-married, and/or a parent with many children were dominant characteristics within the less-complying groups of society in the initial stage of the pandemic. Interestingly, our findings also suggest that rising temperature levels increased non-compliance of people with these individual characteristics.

For countries where herd immunity through vaccination is not yet achieved, the adherence to lockdown and/or social distancing measures may remain an important tool to successfully contain this global pandemic as it enfolds. As the fast spread of Omicron has shown, governments have in many cases been forced to re-adopt and/or adapt social distancing measures—even in countries with relatively high vaccination rates—to prevent overload of the capacity of their public healthcare systems. In other words, while vaccination is arguably the most effective instrument in the fight against the virus, punctual restrictions on social distancing (or other public health measures) may still be required in the future to respond effectively to new strains of the virus. Thus, although warmer periods may reduce the epidemiological potency of the virus, the associated sociologically driven increases in social gatherings and non-compliance with public health rules, particularly present among certain socioeconomic groups identified in this article, may complicate governments’ containment efforts.

Therefore, policymakers around the world are well advised to take the higher likelihood of non-compliance of certain socioeconomic groups on warm and sunny seasons into account when choosing between competing strategies. Specifically, if future intensifying rates of infection require the imposition of lockdown measures, governments should provide additional social assistance to groups for which social distancing entail particularly large financial and non-financial costs (e.g. large families, unemployed, amongst others). More targeted vaccination campaigns and communication strategies may also be warranted. This is especially important as the individual characteristics associated with non-compliance in this article appear very similar to those associated with vaccine hesitancy identified in recent contributions^20^. Thus, combating this pandemic in the long run will necessitate developing effective strategies to convince these groups to vaccinate and comply with public health measures.

## Methods

### Data

Our empirical analysis relies on a survey dataset compiled by YouGov. It consists of 111,694 respondents in total in the UK surveyed between 23rd of March and 18th of May 2020. YouGov has a global online panel of 14 million people, which it has used to run extensive surveys of people in countries affected by covid-19. Significant portions of these covid-specific surveys are now available to the academic community, including a 30-country behavior tracker in partnership with Imperial College London^24^. There is a burgeoning literature using these YouGov covid-19 surveys, for instance elderly people’s response to covid-19^25^.

For the present article, we were granted access to the YouGov survey which consists of 105,512 individuals (once the variables are recoded), fielded daily to a nationally representative sample over a 55-day period between March and May 2020. Such a large representative sample size is very rare in the world of social science surveys because the prohibitive costs required to run such large-scale data collection exercise in one single country. For comparison, the European Social Survey typically covers between 1000 and 3000 individuals in each country, so achieving large sample sizes then requires pooling many countries and waves.

**Figure 4:**
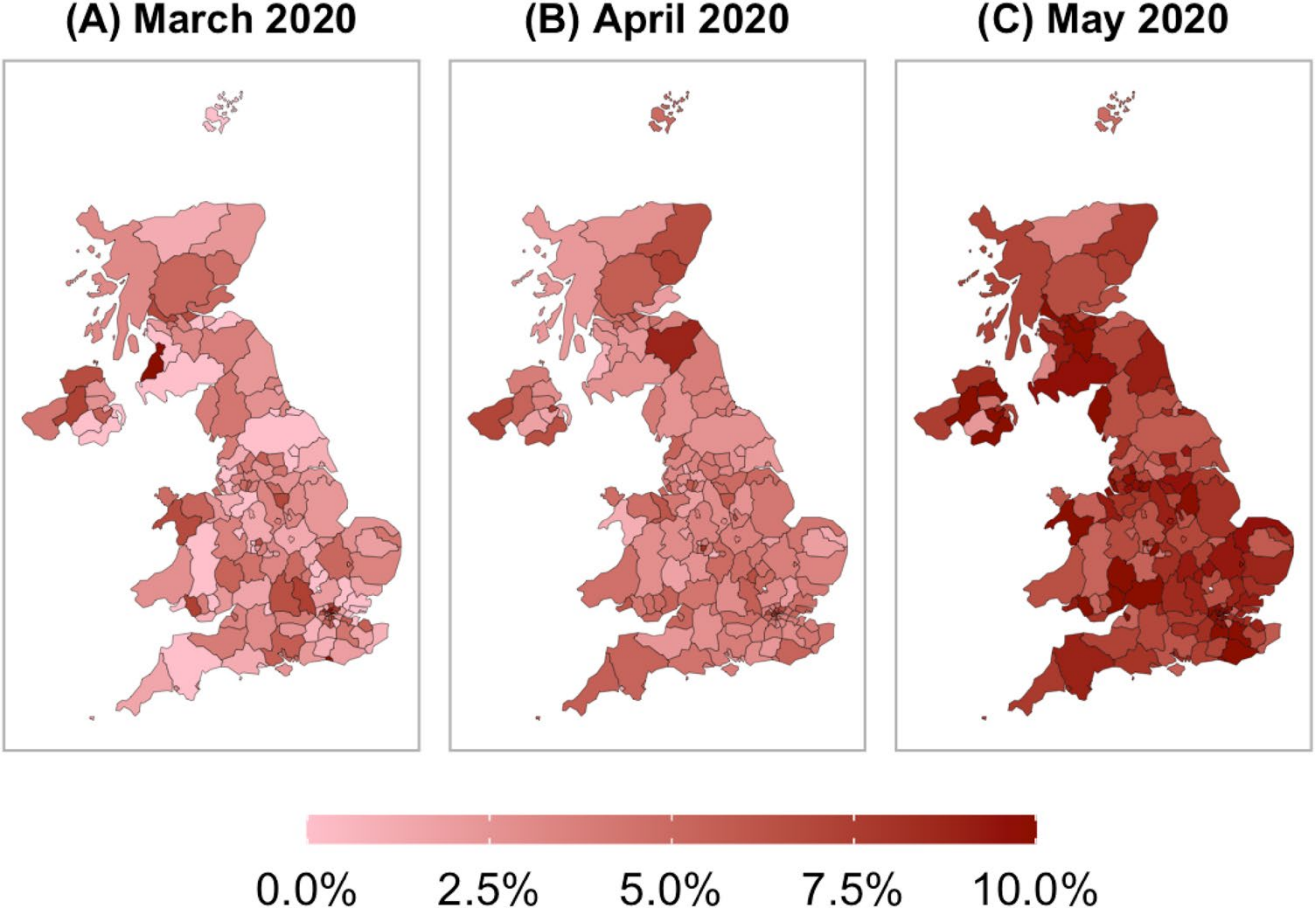
The share of non-compliance varied across regions and over. The panels show the share of non-compliers for each NUTS3 region for different months. In general, non-compliance was the highest in May (**C**) and the lowest in March (**A**), although regional variation exists in all months. Due to these large variations of non-compliance and weather indicators across regions, the inclusion of geographical fixed effects was important to condition on region-specific unobservables. Sample weights are applied. N = 105,512. The maps were created with the programming language R^26^.

Beyond the pure volume of the dataset, the survey provided an unprecedented temporal and geographical granularity (147 regions over 55 days) which was essential for the present research design at hand (Fig. 4). First, the evolution of the pandemic has shown that the situation in a region can change very quickly and lead to large heterogeneity across regions and time periods, even within countries. To account for these confounding factors and in this way isolate individual-level determinants from geographical and time-varying once, the high regional and temporal granularity enabled us to control for different sources of unobserved heterogeneity through appropriate fixed effects. Second, since we are interested in the effect of weather on compliance behavior, our research design required climatological indicators for each survey responses. Here, the detailed information about the location and time when and where the survey was taken enabled us to match high-quality weather variables to each observation in the survey. This was particularly important for the UK where the weather differed substantially across regions and changed quickly even throughout the day.

Our dependent variable of interest was based on the survey question “Which comes closer to describing you?”. This question has three answer options: (A) “'I will probably follow the advice of the government even if I don't agree with it or find it pointless”; (B) “'I will probably do my own thing, regardless of government advice”; and (C) “Don't know”. We transformed this into a binary variable coded 1 if one chooses option (B), and 0 if one chooses option (A). We dropped observations about individuals who choose option (C). In total, 105,512 participants answered this question with (A) or (B).

We cross-checked our non-compliance dummy variable with the (inverted) country-level residential mobility measure provided by Google^19^ (Fig. 1). Although the mobility and compliance did not measure exactly the very same concept, a high correlation between the two should be present if our measure has *prima facie* validity. The Spearman correlation coefficient between the two measures is 0.85. In a linear univariate regression with the aggregated compliance measure as dependent variable and the google mobility indicator as independent variable, the R-squared reaches 0.72. The fact that the inverted residential mobility variable was able to account for large parts of the variation in non-compliance at the national level in the early stage of the pandemic gives us confidence in the validity of our dependent variable. If anything, the potential non-disclosure and social-desirability bias of the survey means our estimates were conservative, thereby underestimating the true share of non-compliance.

However, since the mobility indicator measures behavior at the regional instead of the individual level, we conducted an additional cross-validation test by using individual responses to specific behavioral questions. More specifically, as SI. Fig. 2 shows, respondents who reported that (A) they left home for a longer period, (B) they went to pursue fun activities, or (C) they did not focus extensively on self-isolation, all indicated higher shares of reported non-compliance than their respective peer groups. Thus, since both cross-validation checks at the micro and macro level were in line with our compliance measure, we believe that our dependent variable is a good proxy of actual compliance.

The survey also included information capturing important individual characteristics of the respondents. Based on previous theoretical and empirical work in political science and sociology, we used the following individual level variables: (i) gender (male, female); (ii) age groups (18–24, 25–34, 45–54, 55–65, 65 +); (iii) education level (high, medium, low); (iv) social grade (AB, C1, C2, DE); (v) marital status (married, not married, divorced, widowed); (vi) number of children (0, 1, 2, 3 or more); and (vii) employment status (full-time, part-time, unemployed, student, retired and other). The education level variables were aggregates consisting of 18 different degree types. The social grade classification was based on Social Grade system developed by the National Readership Survey. All of our independent variables were recoded as a series of dichotomous variables to facilitate interpretation. While there were further potential indicators included in the dataset that could have been beneficial for the empirical analysis (i.e., partisanship self-identification or income levels), we did not include them in the empirical model because of very limited data coverage.

Finally, we collected highly granular weather indicators (temperature, solar radiation and total precipitation) from the ERA5 dataset from the Copernicus program. The Copernicus project—funded by the European Commission—has collected data on climatology and weather indicators on a near-real-time basis since 1979. The ERA5 dataset provided weather indicators on an hourly basis with a spatial resolution of ~ 28 km-by-28 km near the equator (0.25° × 0.25°). This high granularity at the geographical level was important in order to match valid weather indicators to the exact location of the respondent. With respect to the temporal frequency, we collapsed these hourly weather variables to “day/nighttime” indicators by averaging the hourly values in four groups: (i) morning (06 AM–11 AM); (ii) afternoon (12 AM–5 PM); (iii) evening (6 PM–11 PM) and (iv) night (12 PM–5 AM). Thus, for instance, if a person had started the survey at 14.36 PM, the respondent was matched to the weather indicators averaged for afternoon values of that particular date. SI. Fig. 2B illustrates the distribution of the temperature variable across all survey responses. Although we do not report estimations using the raw hourly weather indicators, our estimates are robust to different ways of weather operationalization and measurement. SI. Table 1 provides additional information on definition, source and summary statistics for all variables used in the empirical analysis.

### Empirical analysis

Our empirical strategy consisted of two steps. In the first, we investigated how individual level factors are generally associated with non-compliance. In the second, we analyzed how the effect size of these socioeconomic determinants differed under various weather conditions. In terms of independent variables, we focused on three weather indicators, while we controlled for eight individual level socio-demographic and socio-economic characteristics: gender, age groups, education, social class, marital status, number of children, and employment status (see distribution among non-compliers in SI. Fig. 3). In our baseline specification, we used a logistic model by regressing the non-compliance measures on the individual level characteristics of the respondents in the following form:

1 $${\text{Log}}\left( {\frac{{p_{i} }}{{1 - p_{i} }}} \right) = {\upbeta }_{0} + {{ \upbeta }}_{k} {\text{X}}_{k,i} + {{ \upalpha }}_{{\text{r}}} + {\upgamma }_{{\text{t}}} + {\upvarepsilon }_{i}$$

where *p*_*i*_ is the probability of an individual *i* not complying; *X* is a matrix of k individual level variables; α_r_ and γ_t_ are region and date fixed effects, respectively, in region *r* and date *t*; and ε_*i*_ is an individual-specific error term. The regional and date fixed effects are included to control for unobserved cross-regional and temporal heterogeneity. This enabled us to control for confounding factors at the date and regional levels, which might have influenced non-compliance behavior at the time of the survey. In this way, we aimed to limit omitted variable bias and related endogeneity concerns. This is particularly important given the large increase in non-compliance after the announcement of the Prime Minister to ease lockdown rules. By including date fixed effects, we de-meaned the dependent variable by its daily national average and in this way mitigated the risk of potential confounding factors.

### Robustness and sensitivity analysis

Moreover, we tested whether our findings are robust to different fixed effect structures. The results do not dependent on the in- or exclusion of temporal or regional fixed effects (SI. Table 2, columns 1–4). Another concern might have been that confounding factors within regions at a certain period were driving non-compliance behavior, such as external shock (i.e. abrupt virus spread) that only mattered to a certain region at a specific time. Including regional and date fixed effects individually would not have absorbed this variation as they estimated average effects for a given region (over the whole period) or a particular date (across all regions). Thus, we addressed this issue by including NUTS1-Date fixed effects (interaction between date and NUTS1 binaries) and NUTS2-week fixed effects (interaction between week and NUTS2 binaries). Again, our estimates are very similar to the baseline results (SI. Table 2, columns 5–6).

One might also be interested whether the variation in non-compliance was affected by the local pandemic situation and whether the inclusion of individual-level characteristics provided value in terms of goodness of fit. Since general R-Squared might not be a suitable candidate for measuring goodness of fit, we resort to the Akaike Information Criteria (AIC). As SI. Table 2 shows, all estimations reached AIC values of ~ 39,000. When we dropped all individual-level independent variables and only included NUTS1-Date fixed effects (interaction between date and NUTS1 binaries) (as in column 5), the AIC increased to over 42,000. In other words, including individual-level covariates enhanced the goodness of fit and the explanatory power of our control variables seemed to matter after local time-varying factors (such as number of new cases) have been accounted for.

In addition, since the baseline uses standard errors clustered at the regional level, we also ran robustness checks by using different standard error types, namely (i) uncorrected, (ii) robust, (iii) robust clustered at region level, (iv) robust clustered at region-week level, and (v) robust clustered at region-date level. Our results showed that the size of standard errors of the coefficients for different independent variables did not substantially vary depending on the type of standard error that we report (see SI. Table 3).

Furthermore, another concern was related to “double measurement” of the same factor on the right-hand-side. As is common for micro-level survey analysis, demographic and socio-economic variables are usually correlated (i.e., social class, employment status and/or education level), which would bias our estimates and foster model uncertainty. Although none of our independent variables were highly correlated with each other and none of the independent variables had a variance inflated factor (VIF) larger than 5 in the baseline estimation, we show that our results are not subject to multicollinearity issues by including the independent variables in a stepwise fashion (SI. Table 4). Similarly, including a time-trend did not change our findings. In addition, it was also noteworthy that we did not use partisan alignment and income as independent variables in any estimations although they seemed to be quite important given the large variation observable in SI. Fig. 3. This is because the number of observations for income was too low which would have created sampling and endogeneity issues, which are hard to address within the models itself. However, the variation in compliance behavior was remarkable for income. The association between compliance seemed to be highly non-linear with the poorest and richest being least compliant.

We also varied the estimation model by re-running our analyses using a linear probability model with region and date fixed effect:

2 $${\text{y}}_{i} = {\upbeta }_{0} + {{ \upbeta }}_{k} {\text{X}}_{k,i} + {{ \upalpha }}_{{\text{r}}} + {\upgamma }_{{\text{t}}} + {\upvarepsilon }_{i}$$

where y_*i*_ is the non-compliance binary of individual *i*; *X* is a matrix of k individual level variables; α_r_ and γ_t_ are region and date fixed effects, respectively; ε_*i*_ is an individual-specific error term.

Next, in lieu of a fixed effect model, we chose to specify a multilevel random intercept logistic regression:

3 $${\text{Log}}\left( {\frac{{p_{i} }}{{1 - p_{i} }}} \right) = {\upbeta }_{0} + {{ \upbeta }}_{k} {\text{X}}_{k,i} + {{ \upgamma }}_{{\text{t}}} + {\text{u}}_{{\text{r}}} + {\upvarepsilon }_{i}$$

where *p*_*i*_ is the probability of an individual i not complying; *X* is a matrix of k individual level variables; γ_t_ are date fixed effects; u_r_ is the region-specific random intercept; ε_*i*_ is an individual-specific error term. This multilevel mixed-effect specification enabled us to predict the probability of non-compliance of individual i through individual-level characteristics that were nested in different regions.

In the presence of hierarchical structures, standard logistic or linear estimations tend to underestimate the standard errors and thus overestimate the corresponding p-values of predictors. Since there are good reasons to believe that the unit of analysis—in our case survey responses—were not completely independent but rather clustered at the geographical level (i.e., due to neighborhood effects), testing the robustness of our results with a multilevel mixed effect estimation is important. Finally, we also estimated a multi-level mixed-effect generalized linear model with date fixed effects and regional random effects included. As SI. Table 5 shows, the results were very similar in terms of effects size, significance level and the general conclusion we draw from our baseline model.

Finally, to test the mediating effects of weather conditions on the non-compliance behavior of certain groups, we adopted a sub-sampling strategy by estimating the baseline model over sub-samples defined along thresholds in the distribution of our weather variables. To be more specific, for temperature (Fig. 3) and solar radiation (SI. Fig. 5), we restricted the full dataset to observations that are (i) below and (ii) above the first and third quartile of the weather variable, respectively. For precipitation, we created a sample consisting of observations which had (i) zero and (ii) non-zero values for rainfall (SI. Fig. 6). This yielded us six sub-samples to which we applied our baseline model.

## Supplementary Information

Supplementary Information.

## Data Availability

All data and code used in the empirical analysis of the main text or the supplementary material are available upon request from the authors.
