# Supplementary material for: Compliance with the first UK covid-19 lockdown and the compounding effects of weather
Source: Sci Rep. 2022 Mar 9;12:3821. doi: 10.1038/s41598-022-07857-2 (PMC8907269; doi:10.1038/s41598-022-07857-2)
Supplement: Supplementary file 1 — Supplementary Information. [file 41598_2022_7857_MOESM1_ESM.docx]

**Compliance with the first UK covid-19 lockdown and the compounding effects of weather**

Supplementary Information

**Michael Ganslmeier^1^**, **Jonathan Van Parys^2^, Tim Vlandas^3*^**

1 University of Oxford.
Address: University of Oxford, 32 Wellington Square, OX1 2ER Oxford, United Kingdom
Email: michael.ganslmeier@spi.ox.ac.uk

2 YouGov.
Address: YouGov, 50 Featherstone Street, London, EC1Y 8RT, United Kingdom
Email: jonathan.vanparys@yougov.com

3 University of Oxford.
Address: University of Oxford, 32 Wellington Square, OX1 2ER Oxford, United Kingdom
Email: tim.vlandas@spi.ox.ac.uk

# SI. table 1. Definition, source and summary statistics of variables

| **Variable** | **Definition** | **Source** | **N** | **Mean** | **SD** |
| --- | --- | --- | --- | --- | --- |
| noncompliance | Not complying | YouGov | 105,512 | 0.0518 | 0.222 |
| male | male | YouGov | 105,512 | 0.466 | 0.499 |
| age1824 | age: 18 and 24 | YouGov | 105,512 | 0.0936 | 0.291 |
| age2534 | age: 25 and 34 | YouGov | 105,512 | 0.156 | 0.363 |
| age3544 | age: 35 and 44 | YouGov | 105,512 | 0.166 | 0.372 |
| age4554 | age: 45 and 54 | YouGov | 105,512 | 0.161 | 0.367 |
| age5564 | age: 55 and 65 | YouGov | 105,512 | 0.165 | 0.371 |
| age65 | age 65 or over | YouGov | 105,512 | 0.258 | 0.438 |
| socgradeAB | Upper middle and middle class | YouGov | 104,597 | 0.303 | 0.460 |
| socgradeC1 | Lower middle class | YouGov | 104,597 | 0.295 | 0.456 |
| socgradeC2 | Skilled working class | YouGov | 104,597 | 0.185 | 0.388 |
| socgradeDE | Working and non-working class | YouGov | 104,597 | 0.218 | 0.413 |
| eduHigh | high education | YouGov | 105,512 | 0.305 | 0.460 |
| eduMed | medium education | YouGov | 105,512 | 0.432 | 0.495 |
| eduLow | low education level | YouGov | 105,512 | 0.264 | 0.441 |
| maritalMarried | married | YouGov | 104,831 | 0.610 | 0.488 |
| maritalNotMarried | not married | YouGov | 104,831 | 0.262 | 0.439 |
| maritalDivorced | divorced | YouGov | 104,831 | 0.0881 | 0.283 |
| maritalWidowed | widowed | YouGov | 104,831 | 0.0400 | 0.196 |
| child0 | no children | YouGov | 101,454 | 0.744 | 0.437 |
| child1 | 1 child | YouGov | 101,454 | 0.119 | 0.324 |
| child2 | 2 children | YouGov | 101,454 | 0.1000 | 0.300 |
| child3p | 3 children or more | YouGov | 101,454 | 0.0376 | 0.190 |
| workFullTime | full-time employment | YouGov | 105,512 | 0.396 | 0.489 |
| workPartTime | part-time employment | YouGov | 105,512 | 0.142 | 0.350 |
| workStudent | student | YouGov | 105,512 | 0.0493 | 0.217 |
| workRetired | retired | YouGov | 105,512 | 0.272 | 0.445 |
| workUnemp | unemployed | YouGov | 105,512 | 0.0377 | 0.191 |
| workOther | other work employment | YouGov | 105,512 | 0.102 | 0.303 |
| precipitation | precipitation | Copernicus | 105,506 | 0.00035 | 0.0012 |
| solar_radiation | solar radiation | Copernicus | 105,506 | 5.555 | 4.886 |
| temperature | temperature | Copernicus | 105,506 | 283.3 | 3.444 |


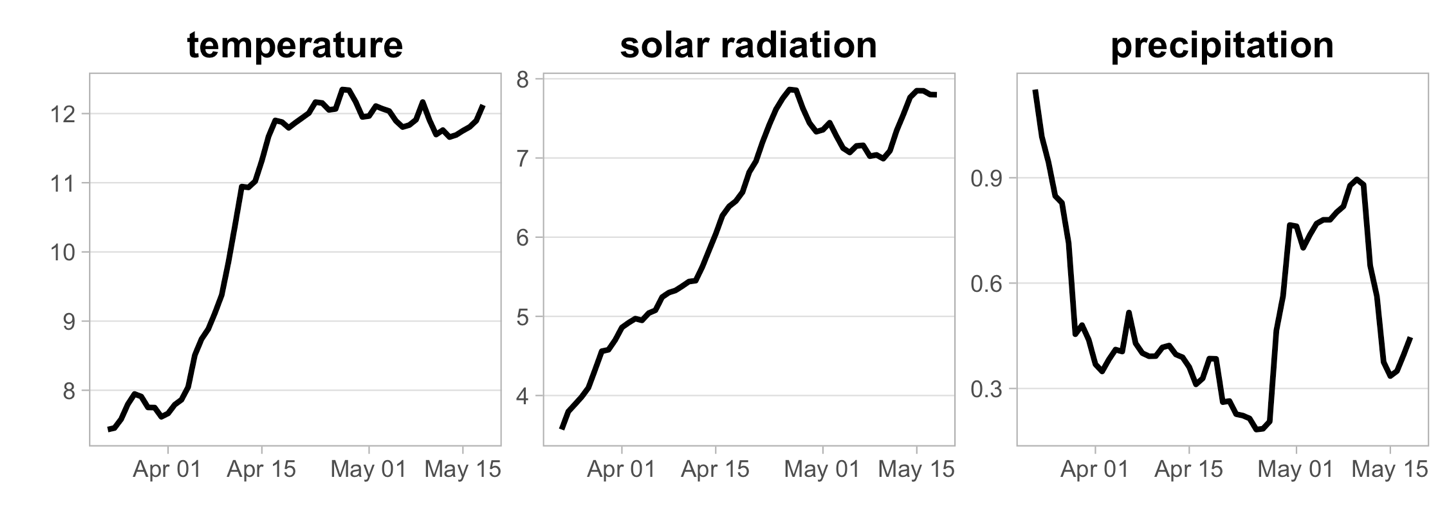


**SI. Fig. 1A. Average weather indicators over time.** The panels show average weather indicators at 12:00AM in the UK between 23^rd^ of March and 18^th^ of May 2020. The units of temperature, solar radiation and precipitation are degrees Celsius, MJ/m² (in Mio.) and m x 10^-7^, respectively. The figure illustrates that temperature levels are strongly increasing in the first weeks of April, similar to solar radiation. Precipitation is more volatile over time.


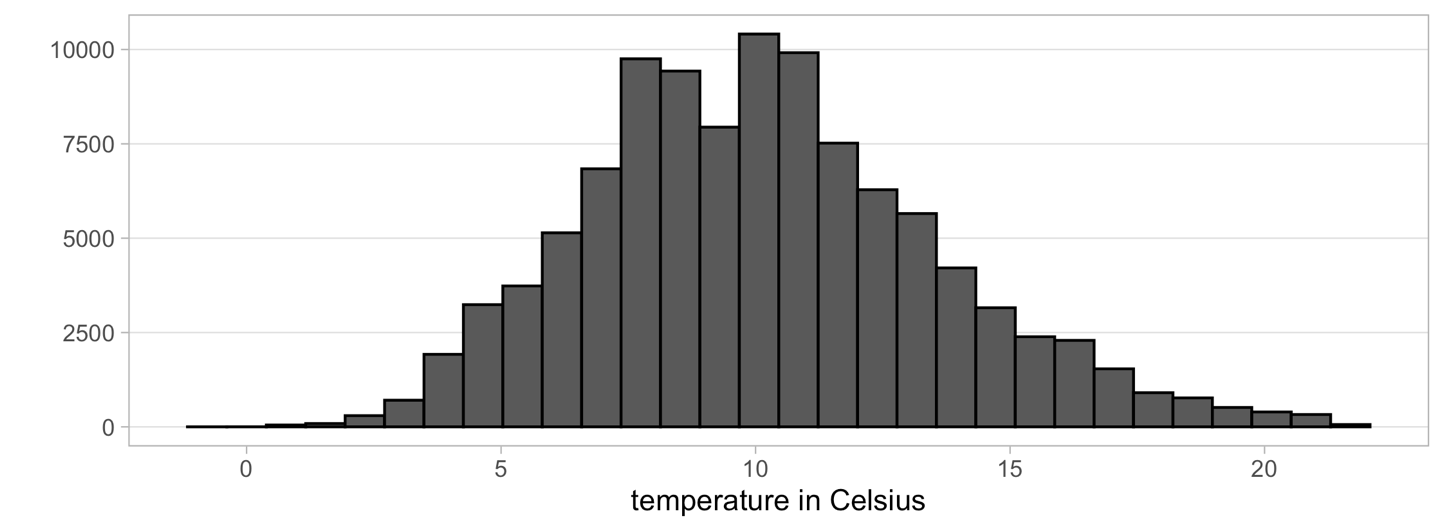


**SI. Fig. 1B. Distribution of temperature values across survey responses.** The figure shows the distribution of temperature values for all responses in the survey dataset. The unit of temperature is degrees Celsius. N = 105,512


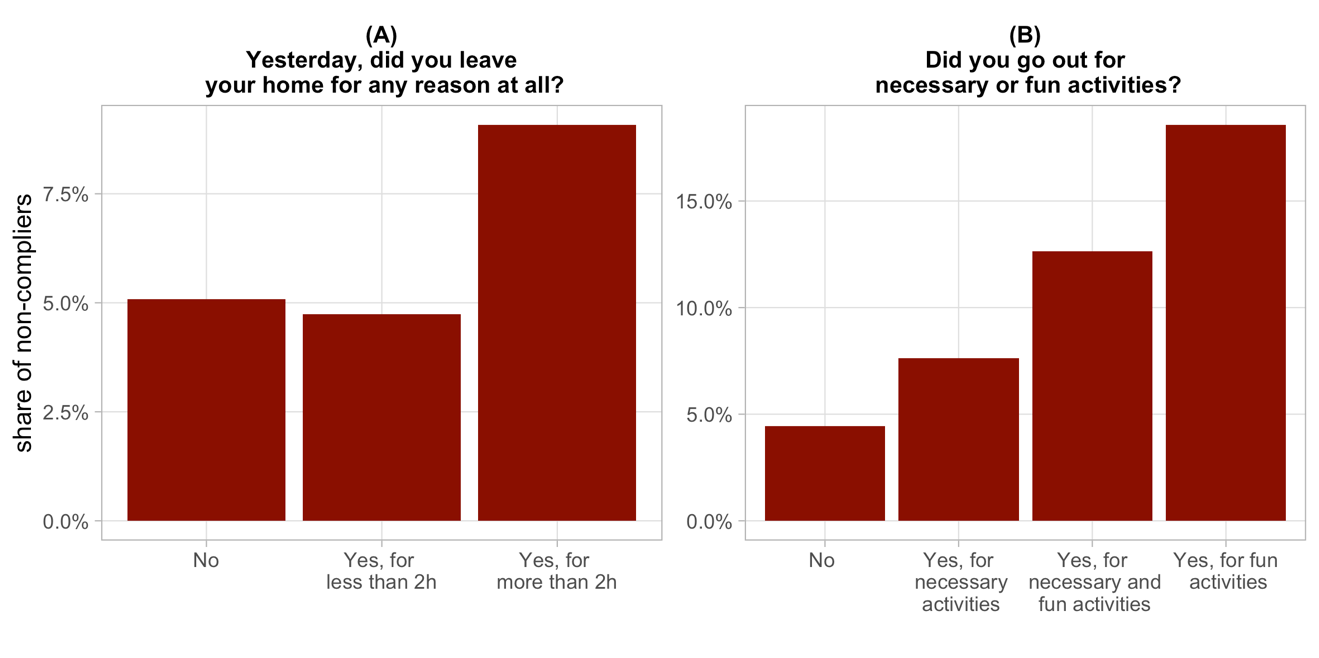

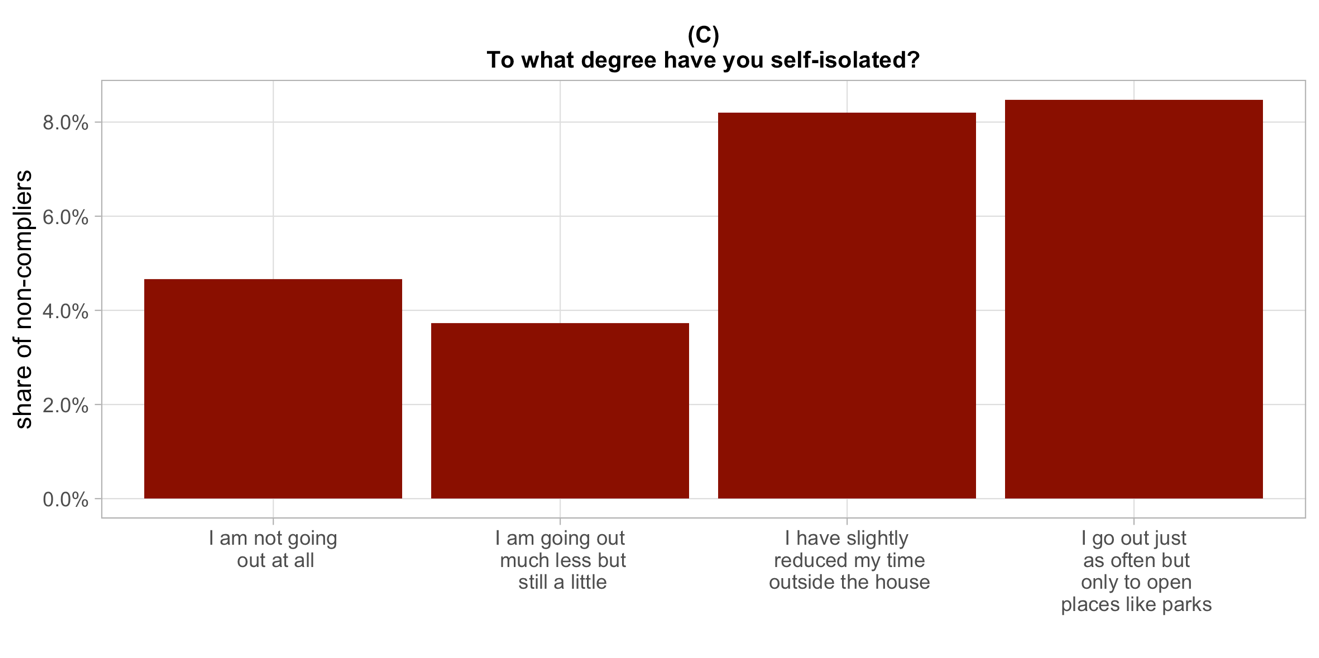


**SI. Fig. 2. Robustness checks of non-compliance by behavioural survey items.** The panels show the share of non-compliers for each answer to question related to actual behaviour. The figure illustrates that people who are self-isolating to a lower degree or engage in more fun activities, they are also more likely to report that they are non-compliers. Sample weights are applied.


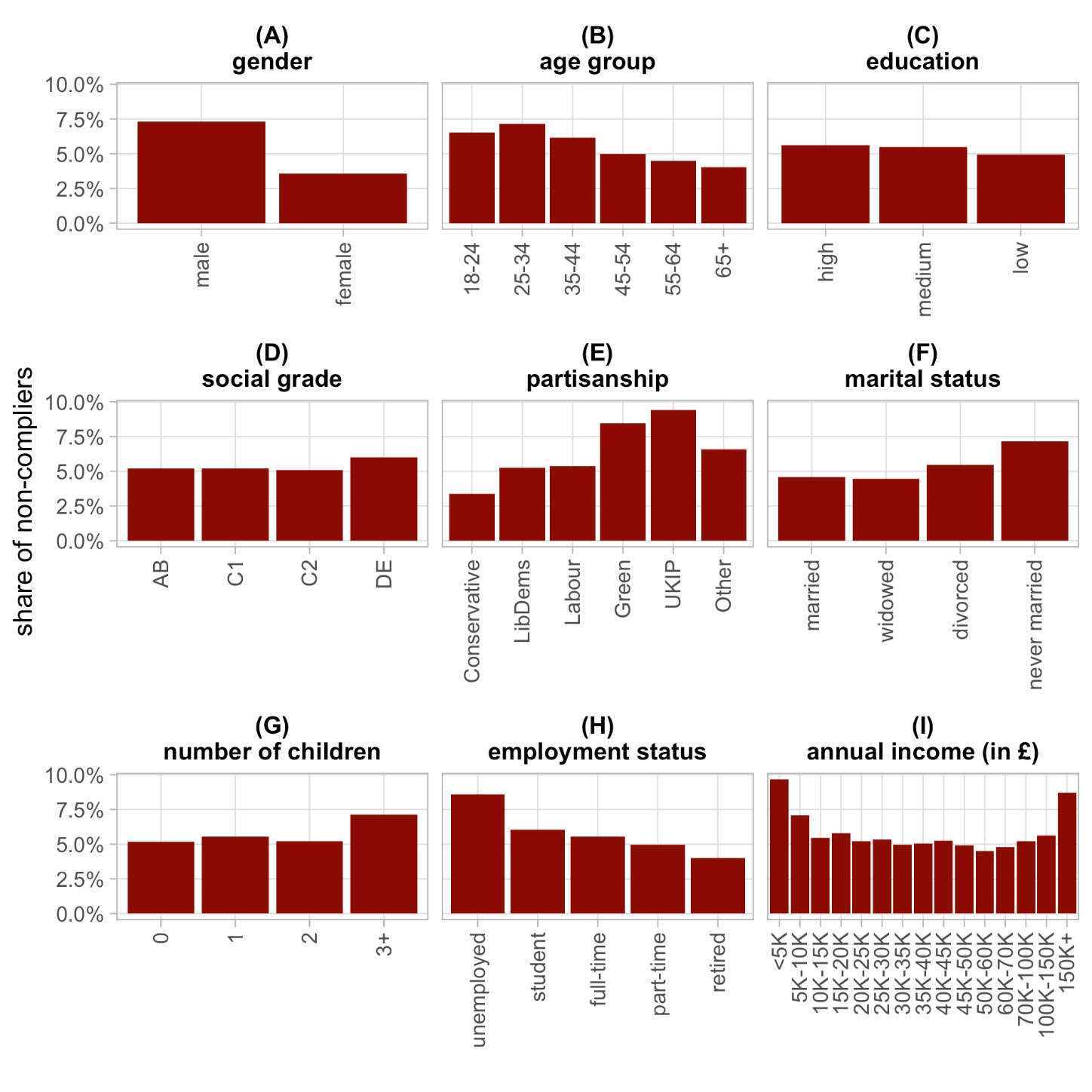


**SI. Fig. 3. The share of non-compliers across individual characteristics.** The panels show the share of non-compliers for each category of selected individual characteristics. The descriptive evidence illustrates that non-compliance is the largest among people who are (i) male, (ii) young, (iii) in very low social class, (iv) green and UKIP voters, (v) never married, (vi) with many children, (vii) unemployed and (viii) with very low or very high income. Sample weights are applied. N = 105,512


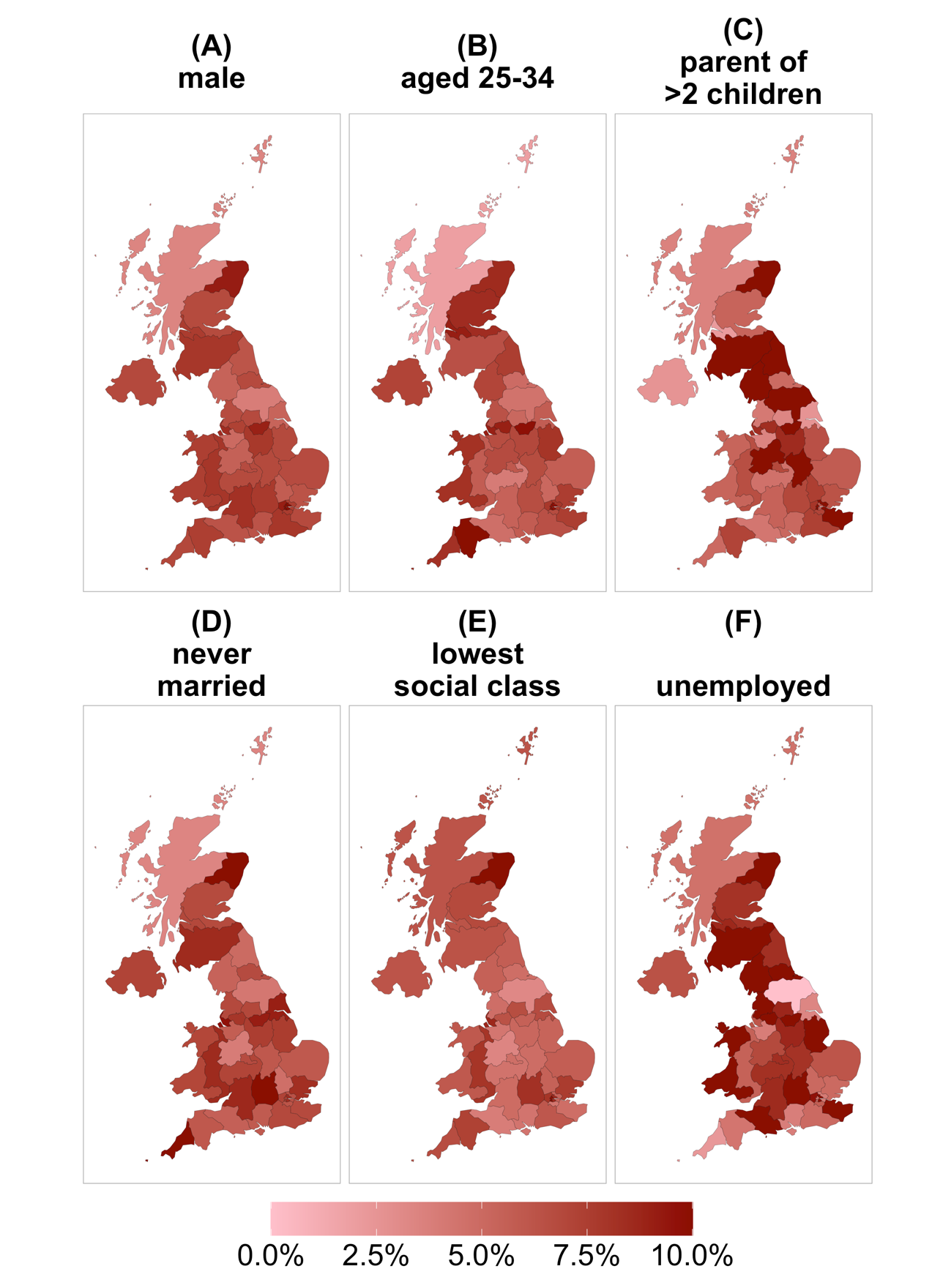


**SI. Fig. 4. Non-compliers by individual characteristics across.** The panels show the share of non-compliers for each NUTS2 region by selected individual characteristics in the whole sample. Parents with more than 2 children and unemployed people show particular high rates of non-compliance in Northern England and Southern Scotland. Sample weights are applied. N = 105,512. The maps were created with the programming language R^24^.

# SI. table 2. Logistic regressions with different fixed effect structures

| **Model** | **(1)** | **(2)** | **(3)** | **(4)** | **(5)** | **(6)** |
| --- | --- | --- | --- | --- | --- | --- |
| male | 0.793*** | 0.797*** | 0.798*** | 0.802*** | 0.803*** | 0.800*** |
| age: 18-24 | 0.125 | 0.101 | 0.136 | 0.115 | 0.097 | 0.116 |
| age: 25-34 | 0.418*** | 0.424*** | 0.428*** | 0.434*** | 0.426*** | 0.424*** |
| age: 35-44 | 0.252*** | 0.256*** | 0.264*** | 0.270*** | 0.258*** | 0.264*** |
| age: 45-54 | 0.046 | 0.043 | 0.059 | 0.057 | 0.040 | 0.055 |
| age: 55-64 | -0.024 | -0.009 | -0.014 | 0.003 | -0.004 | 0.001 |
| social grade C1 | -0.055 | -0.059 | -0.053 | -0.058 | -0.053 | -0.060 |
| social grade C2 | -0.022 | -0.033 | -0.006 | -0.019 | -0.017 | -0.017 |
| social grade DE | 0.038 | 0.041 | 0.045 | 0.050 | 0.059 | 0.045 |
| low education | -0.158*** | -0.166*** | -0.152*** | -0.160*** | -0.165*** | -0.153*** |
| medium education | -0.022 | -0.025 | -0.010 | -0.012 | -0.023 | -0.008 |
| never married | 0.325*** | 0.338*** | 0.299*** | 0.312*** | 0.324*** | 0.307*** |
| divorced | 0.344*** | 0.353*** | 0.341*** | 0.347*** | 0.343*** | 0.341*** |
| widowed | 0.307*** | 0.301*** | 0.313*** | 0.306*** | 0.296*** | 0.310*** |
| 1 child | 0.031 | 0.030 | 0.039 | 0.038 | 0.036 | 0.049 |
| 2 children | -0.007 | -0.007 | 0.007 | 0.009 | 0.008 | 0.014 |
| >2 children | 0.291*** | 0.285*** | 0.298*** | 0.295*** | 0.285*** | 0.288*** |
| part-time work | 0.197*** | 0.193*** | 0.202*** | 0.199*** | 0.191*** | 0.201*** |
| student | 0.076 | 0.061 | 0.076 | 0.058 | 0.071 | 0.059 |
| retired | 0.025 | 0.020 | 0.050 | 0.047 | 0.019 | 0.044 |
| unemployed | 0.514*** | 0.452*** | 0.521*** | 0.459*** | 0.445*** | 0.466*** |
| other work status | 0.467*** | 0.452*** | 0.473*** | 0.457*** | 0.449*** | 0.463*** |
| constant | -3.700*** | -4.541*** | -3.733*** | -4.594*** | -3.062*** | -2.975*** |
| Observations | 100,357 | 100,357 | 100,357 | 100,357 | 98,687 | 99,917 |
| Region FE | No | No | Yes | Yes | No | Yes |
| Date FE | No | Yes | No | Yes | No | No |
| NUTS1-Date FE | No | No | No | No | Yes | No |
| NUTS2-Week FE | No | No | No | No | No | Yes |
| AIC | 39,263 | 38,021 | 38,998 | 38,998 | 38,998 | 38,998 |

The outcome variable is the non-compliance binary. The columns differ with respect to the fixed effects (FE) included. Estimates are based on a logistic regression robust standard errors clustered at the regional level. ***,**,* denote significance at 1, 5 and 10 percent, respectively.

| **Model** | **(1)** | **(2)** | **(3)** | **(4)** | **(5)** |
| --- | --- | --- | --- | --- | --- |
| male | 0.802*** | 0.802*** | 0.802*** | 0.802*** | 0.802*** |
| age: 18-24 | 0.115 | 0.115 | 0.115 | 0.115 | 0.115 |
| age: 25-34 | 0.434*** | 0.434*** | 0.434*** | 0.434*** | 0.434*** |
| age: 35-44 | 0.270*** | 0.270*** | 0.270*** | 0.270*** | 0.270*** |
| age: 45-54 | 0.057 | 0.057 | 0.057 | 0.057 | 0.057 |
| age: 55-64 | 0.003 | 0.003 | 0.003 | 0.003 | 0.003 |
| social grade C1 | -0.058 | -0.058 | -0.058 | -0.058 | -0.058 |
| social grade C2 | -0.019 | -0.019 | -0.019 | -0.019 | -0.019 |
| social grade DE | 0.050 | 0.050 | 0.050 | 0.050 | 0.050 |
| low education | -0.160*** | -0.160*** | -0.160*** | -0.160*** | -0.160*** |
| medium education | -0.012 | -0.012 | -0.012 | -0.012 | -0.012 |
| never married | 0.312*** | 0.312*** | 0.312*** | 0.312*** | 0.312*** |
| divorced | 0.347*** | 0.347*** | 0.347*** | 0.347*** | 0.347*** |
| widowed | 0.306*** | 0.306*** | 0.306*** | 0.306*** | 0.306*** |
| 1 child | 0.038 | 0.038 | 0.038 | 0.038 | 0.038 |
| 2 children | 0.009 | 0.009 | 0.009 | 0.009 | 0.009 |
| >2 children | 0.295*** | 0.295*** | 0.295*** | 0.295*** | 0.295*** |
| part-time work | 0.199*** | 0.199*** | 0.199*** | 0.199*** | 0.199*** |
| student | 0.058 | 0.058 | 0.058 | 0.058 | 0.058 |
| retired | 0.047 | 0.047 | 0.047 | 0.047 | 0.047 |
| unemployed | 0.459*** | 0.459*** | 0.459*** | 0.459*** | 0.459*** |
| other work status | 0.457*** | 0.457*** | 0.457*** | 0.457*** | 0.457*** |
| constant | -4.594*** | -4.594*** | -4.594*** | -4.594*** | -4.594*** |
| Observations | 100,357 | 100,357 | 100,357 | 100,357 | 100,357 |
| Region FE | Yes | Yes | Yes | Yes | Yes |
| Date FE | Yes | Yes | Yes | Yes | Yes |
| Standard Error | standard | robust | Clustered at region | Clustered at  region-week | Clustered at  region-date |

# SI. table 3. Logistic regressions with different standard error types

The outcome variable is the non-compliance binary. The columns differ with respect to the type of the standard error. Region and date fixed effects (FE) are included. Estimates are based on a logistic regression. ***,**,* denote significance at 1, 5 and 10 percent, respectively.

| **Model** | **(1)** | **(2)** | **(3)** | **(4)** | **(5)** | **(6)** |
| --- | --- | --- | --- | --- | --- | --- |
| male | 0.751*** | 0.748*** | 0.753*** | 0.758*** | 0.802*** | 0.802*** |
| age: 18-24 | 0.424*** | 0.390*** | 0.220*** | 0.157* | 0.115 | 0.115 |
| age: 25-34 | 0.592*** | 0.567*** | 0.518*** | 0.469*** | 0.434*** | 0.434*** |
| age: 35-44 | 0.412*** | 0.394*** | 0.376*** | 0.319*** | 0.270*** | 0.270*** |
| age: 45-54 | 0.189*** | 0.189*** | 0.170** | 0.118* | 0.057 | 0.057 |
| age: 55-64 | 0.095* | 0.096* | 0.092* | 0.076 | 0.003 | 0.003 |
| social grade C1 | -0.053 | -0.033 | -0.064 | -0.059 | -0.058 | -0.058 |
| social grade C2 | -0.017 | 0.021 | 0.002 | -0.000 | -0.019 | -0.019 |
| social grade DE | 0.227*** | 0.272*** | 0.196*** | 0.166*** | 0.050 | 0.050 |
| low education |  | -0.153*** | -0.149*** | -0.136*** | -0.16*** | -0.160*** |
| medium education |  | 0.008 | 0.006 | -0.003 | -0.012 | -0.012 |
| never married |  |  | 0.319*** | 0.340*** | 0.312*** | 0.312*** |
| divorced |  |  | 0.341*** | 0.350*** | 0.347*** | 0.347*** |
| widowed |  |  | 0.301*** | 0.295*** | 0.306*** | 0.306*** |
| 1 child |  |  |  | 0.051 | 0.038 | 0.038 |
| 2 children |  |  |  | 0.023 | 0.009 | 0.009 |
| >2 children |  |  |  | 0.327*** | 0.295*** | 0.295*** |
| part-time work |  |  |  |  | 0.199*** | 0.199*** |
| student |  |  |  |  | 0.058 | 0.058 |
| retired |  |  |  |  | 0.047 | 0.047 |
| unemployed |  |  |  |  | 0.459*** | 0.459*** |
| other work status |  |  |  |  | 0.457*** | 0.457*** |
| time trend |  |  |  |  |  | 0.032*** |
| constant | -4.565*** | -4.541*** | -4.588*** | -4.552*** | -4.594*** | -4.626*** |
| Observations | 104,597 | 104,597 | 103,944 | 100,357 | 100,357 | 100,357 |
| Region FE | Yes | Yes | Yes | Yes | Yes | Yes |
| Date FE | Yes | Yes | Yes | Yes | Yes | Yes |

# SI. table 4. Logistic regressions with different control variables

The outcome variable is the non-compliance binary. The columns differ with respect to inclusion of independent variables. Estimates are based on a logistic regression robust standard errors clustered at the regional level. Region and date fixed effects (FE) are included. ***,**,* denote significance at 1, 5 and 10 percent, respectively.

| male | 0.802*** | 0.037*** | 0.799*** | 0.037*** |
| --- | --- | --- | --- | --- |
| age: 18-24 | 0.115 | 0.005 | 0.108 | 0.004 |
| age: 25-34 | 0.434*** | 0.022*** | 0.428*** | 0.022*** |
| age: 35-44 | 0.270*** | 0.013*** | 0.261*** | 0.013*** |
| age: 45-54 | 0.057 | 0.003 | 0.049 | 0.003 |
| age: 55-64 | 0.003 | 0.000 | -0.002 | -0.000 |
| social grade C1 | -0.058 | -0.002 | -0.057 | -0.002 |
| social grade C2 | -0.019 | -0.000 | -0.022 | -0.000 |
| social grade DE | 0.050 | 0.003 | 0.049 | 0.003 |
| low education | -0.160*** | -0.007*** | -0.164*** | -0.007*** |
| medium education | -0.012 | -0.001 | -0.017 | -0.001 |
| never married | 0.312*** | 0.016*** | 0.319*** | 0.017*** |
| divorced | 0.347*** | 0.016*** | 0.348*** | 0.016*** |
| widowed | 0.306*** | 0.013*** | 0.301*** | 0.013*** |
| 1 child | 0.038 | 0.000 | 0.035 | 0.000 |
| 2 children | 0.009 | -0.001 | 0.005 | -0.002 |
| >2 children | 0.295*** | 0.014*** | 0.291*** | 0.014*** |
| part-time work | 0.199*** | 0.010*** | 0.195*** | 0.010*** |
| student | 0.058 | 0.003 | 0.065 | 0.003 |
| retired | 0.047 | 0.004 | 0.033 | 0.003 |
| unemployed | 0.459*** | 0.029*** | 0.453*** | 0.029*** |
| other work status | 0.457*** | 0.024*** | 0.453*** | 0.024*** |
| constant | -4.594*** | -0.012** | -4.593*** | -0.012** |
| Observations | 100,357 | 100,357 | 100,357 | 100,357 |
| Region FE | Yes | Yes | No | No |
| Date FE | Yes | Yes | Yes | Yes |
| Model | Logistic | Linear  probability | Multi-level logistic | Multi-level  generalized linear |

# SI. table 5. Regression results for different model types

The outcome variable is the non-compliance binary. The columns differ with respect to the regression method applied. All estimations use robust standard errors clustered at the regional level. ***,**,* denote significance at 1, 5 and 10 percent, respectively.

| male | **0.000** | **0.000** | **0.000** | **0.000** | **0.000** | **0.000** |
| --- | --- | --- | --- | --- | --- | --- |
| age: 18-24 | 0.774 | 0.211 | 0.425 | 0.834 | 0.357 | 0.464 |
| age: 25-34 | **0.010** | **0.041** | **0.001** | 0.370 | 0.416 | **0.000** |
| age: 35-44 | 0.146 | 0.734 | **0.007** | 0.958 | 0.855 | **0.001** |
| age: 45-54 | 0.969 | 0.804 | 0.677 | 0.168 | 0.520 | 0.601 |
| age: 55-64 | 0.744 | 0.996 | 0.698 | 0.220 | 0.245 | 0.870 |
| social grade C1 | 0.589 | 0.874 | **0.017** | **0.037** | 0.610 | 0.138 |
| social grade C2 | 0.981 | 0.996 | **0.006** | 0.817 | 0.497 | 0.564 |
| social grade DE | 0.426 | 0.269 | 0.198 | 0.840 | 0.927 | 0.306 |
| low education | **0.056** | **0.074** | 0.187 | **0.029** | **0.083** | **0.000** |
| medium education | 0.999 | 0.743 | 0.695 | 0.563 | 0.538 | 0.519 |
| never married | **0.002** | **0.003** | **0.000** | **0.006** | **0.000** | **0.000** |
| divorced | **0.000** | 0.155 | **0.000** | 0.611 | **0.029** | **0.000** |
| widowed | **0.042** | 0.684 | 0.401 | 0.579 | 0.721 | **0.000** |
| 1 child | 0.346 | 0.368 | 0.535 | 0.955 | 0.564 | 0.569 |
| 2 children | 0.602 | 0.692 | 0.605 | 0.712 | 0.715 | 0.716 |
| >2 children | **0.001** | 0.439 | 0.139 | **0.027** | **0.042** | **0.000** |
| part-time work | **0.000** | **0.100** | **0.040** | **0.033** | 0.725 | **0.000** |
| student | 0.776 | 0.859 | 0.498 | 0.227 | 0.784 | 0.411 |
| retired | 0.743 | 0.322 | 0.260 | **0.071** | 0.727 | 0.513 |
| unemployed | **0.001** | **0.000** | **0.014** | **0.000** | 0.185 | **0.000** |
| other work status | **0.000** | **0.000** | **0.000** | **0.000** | **0.003** | **0.000** |
| constant | **0.000** | **0.000** | **0.000** | **0.000** | **0.000** | **0.000** |
| Observations | 25,176 | 23,072 | 22,490 | 25,062 | 7,347 | 89,232 |
| Sample | Low temperature | High temperature | Low  solar rad. | High solar rad. | Absence of rain | Presence of rain |
| Region FE | Yes | Yes | Yes | Yes | Yes | Yes |
| Date FE | Yes | Yes | Yes | Yes | Yes | Yes |

# SI. table 6. Coefficient and p-values of regressions with high and low temperature observations

The outcome variable is the non-compliance binary. The columns differ with respect to the sample being used. The samples are based on weather three different weather indicators. The cells represent the p-values of the respective coefficient. All estimations use robust standard errors clustered at the regional level. **Bold** is applied if the p-value is equal/smaller than 0.1.

**
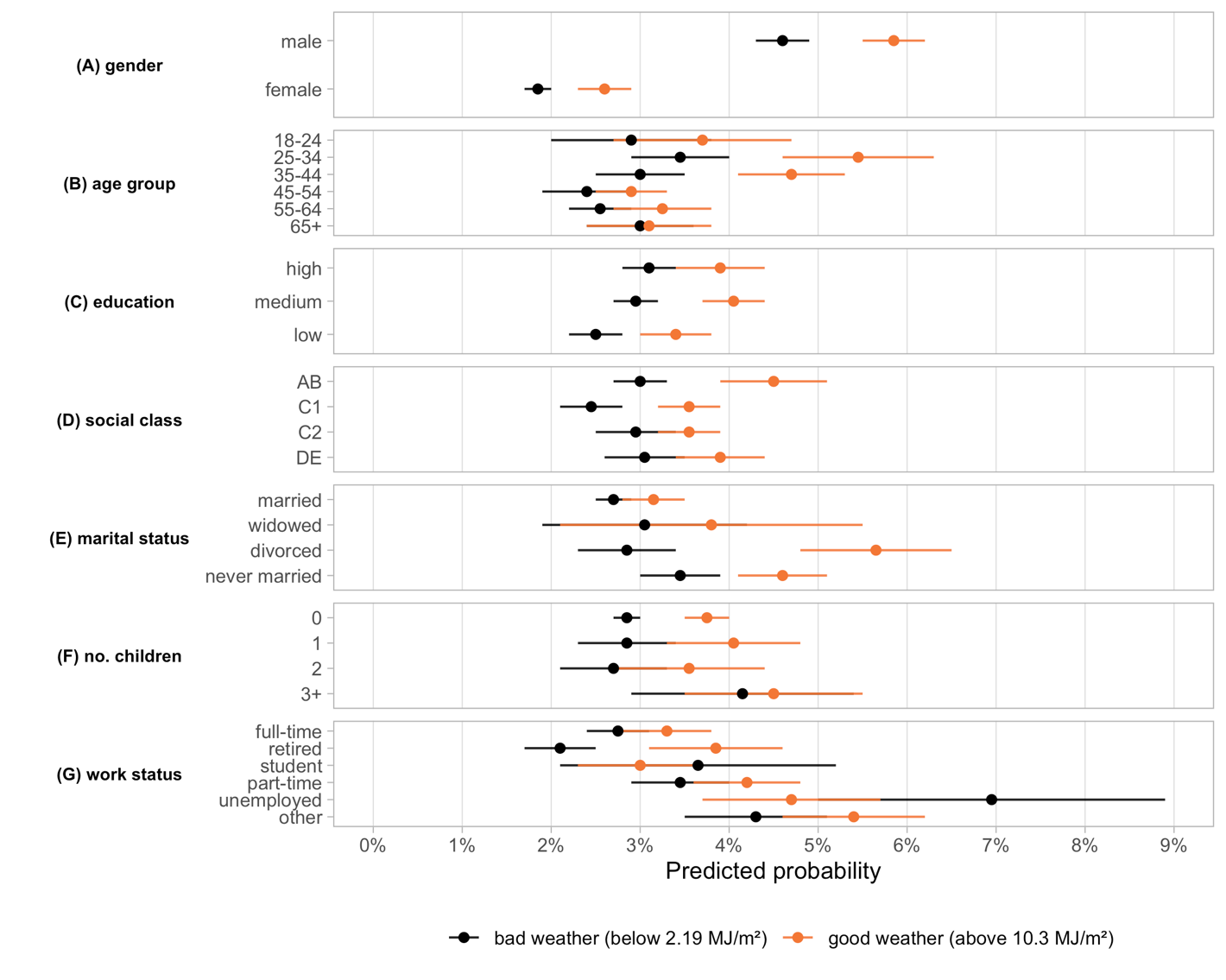
**

**SI. Fig. 5. Predicted probabilities of non-compliance by solar radiation.** The panels show the predicted probability of non-compliance when a given individual level characteristic being equal to 1 while all other covariates are held constant at their mean values. The black (orange) dots are estimates based on observations under bad (good) weather conditions, which are defined along the top and bottom quartiles (2.19 MJ/m2 and 10.3 MJ/m2, respectively) of net solar radiation. The estimates are based on a logistic regression with date and region fixed effects included and robust standard errors clustered at the region level. The horizontal lines show the 95% confidence intervals. N = 100,357.

#
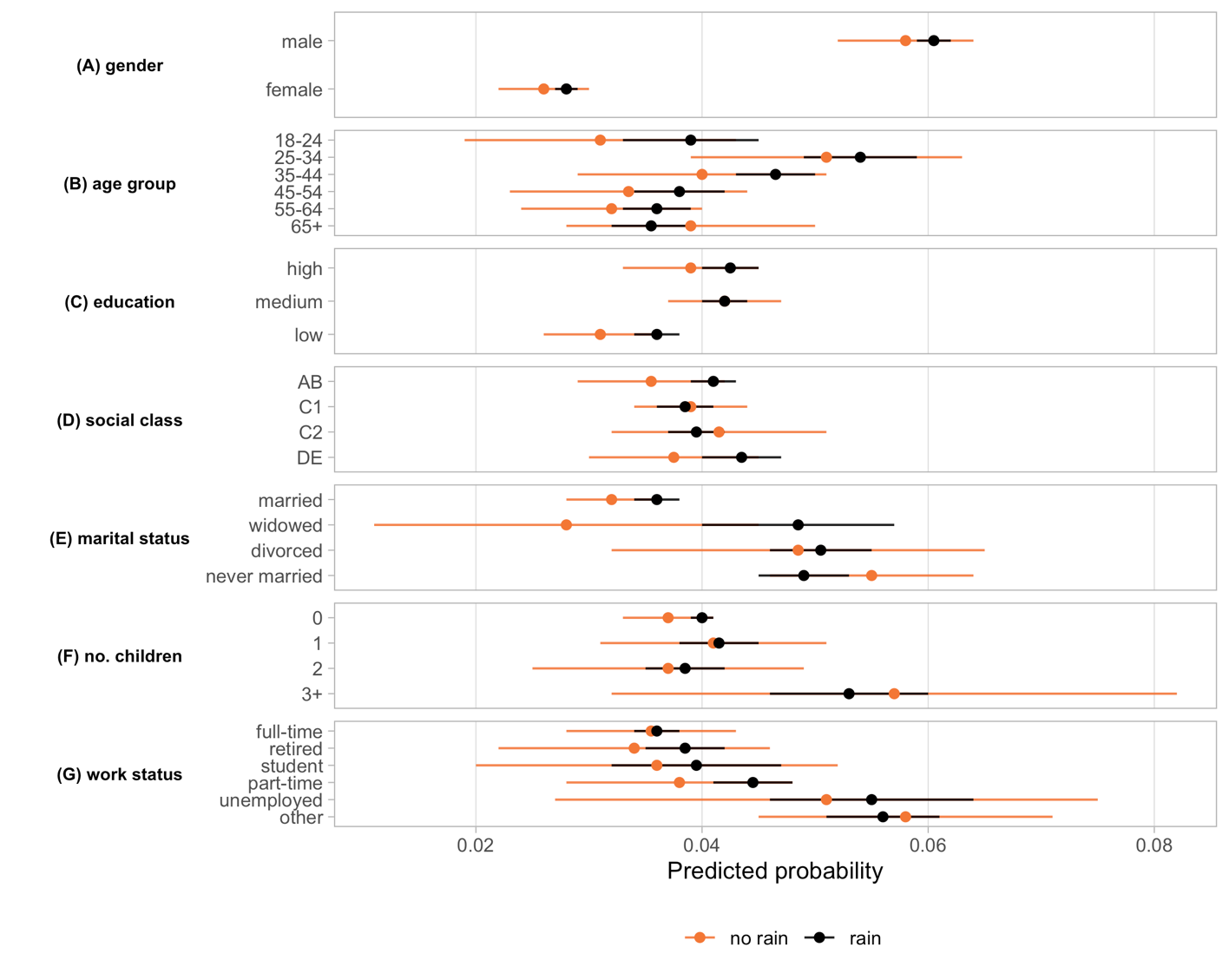


**SI. Fig. 6. Predicted probabilities of non-compliance by precipitation.** The panels show the predicted probability of non-compliance when a given individual level characteristic being equal to 1 while all other covariates are held constant at their mean values. The black (orange) dots are estimates based on observations under bad (good) weather conditions, which are defined along the presence of rainfall. The estimates are based on a logistic regression with date and region fixed effects included and robust standard errors clustered at the region level. The horizontal lines show the 95% confidence intervals. N = 100,357.

#
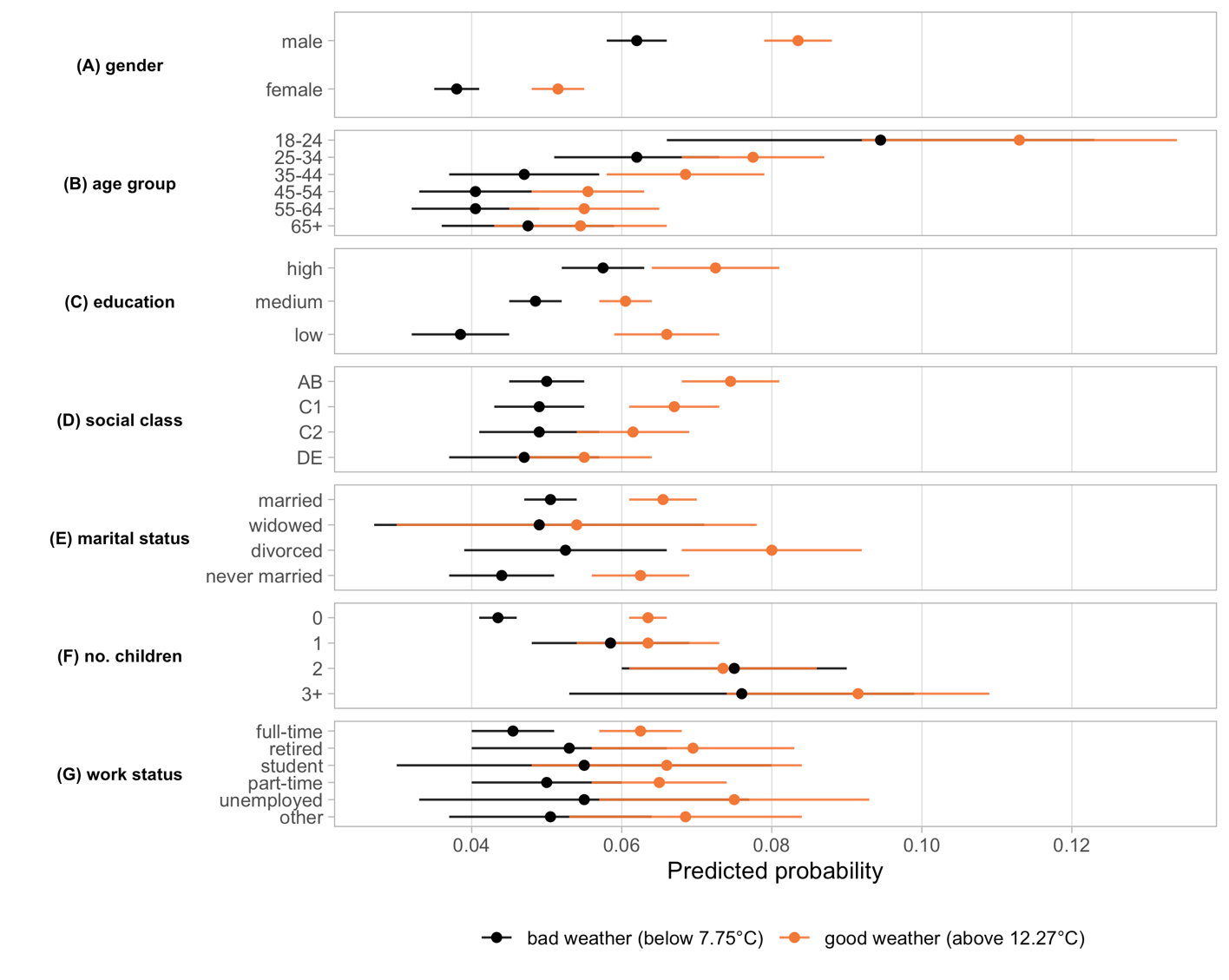


**SI. Fig. 7. Predicted probabilities of going out for fun activities by temperature.** The panels show the predicted probability of going out for fun activities when a given individual level characteristic being equal to 1 while all other covariates are held constant at their mean values. The black (orange) dots are estimates based on observations under bad (good) weather conditions, which are defined along the first and third quartile of the distribution of temperature across all survey responses. The estimates are based on a logistic regression with date and region fixed effects included and robust standard errors clustered at the region level. The horizontal lines show the 95% confidence intervals. N = 100,357.

**
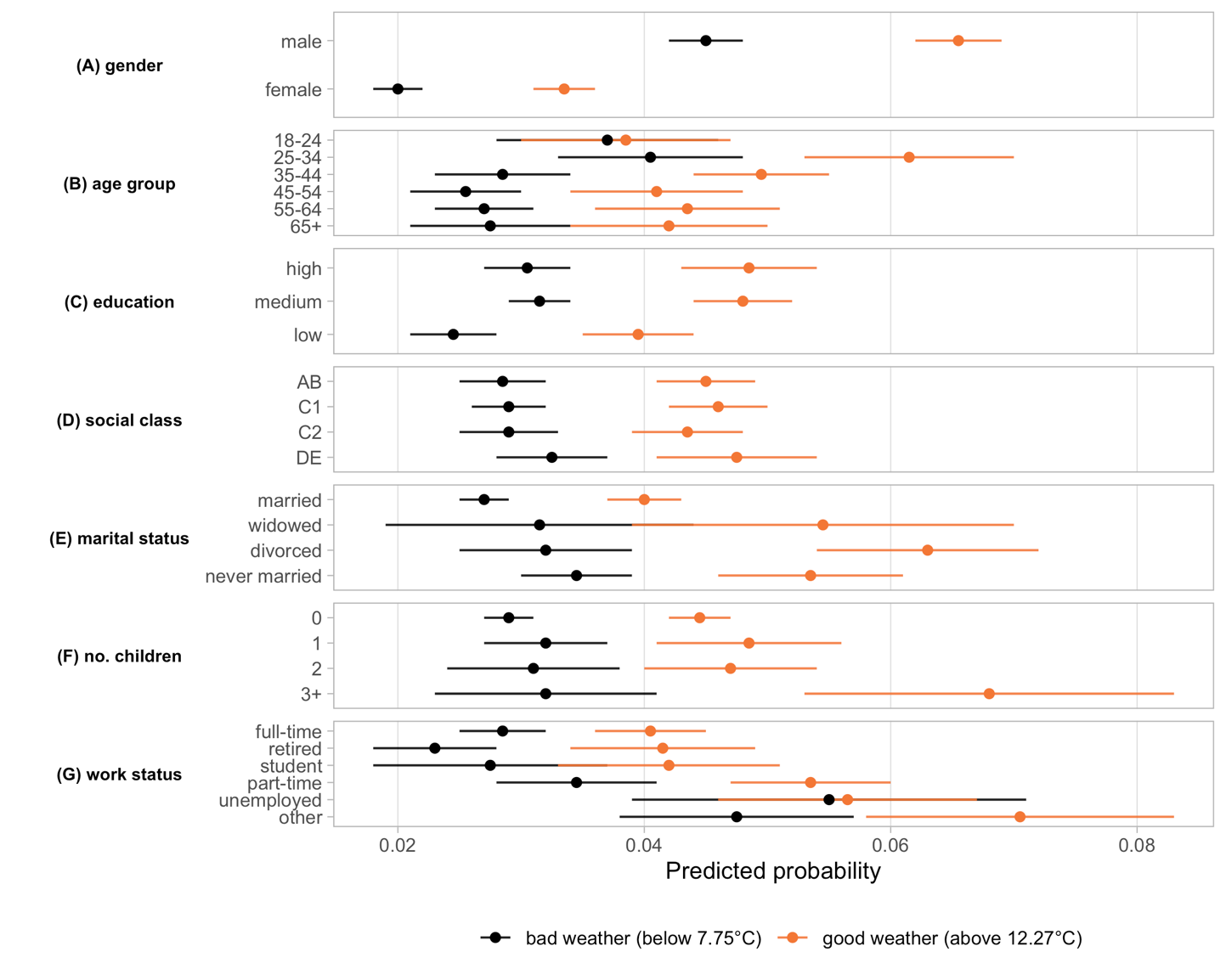
**

**SI. Fig. 8. Warmer temperature has a large effect on non-compliance behavior with certain demographic and socio-economic characteristics.** The panels show the predicted probability of non-compliance when a given individual level characteristic being equal to 1 while all other covariates are held constant at their mean values. The black (orange) dots are estimates based on observations under bad (good) weather conditions, which are defined along the top and bottom quartiles (7.75°C and 12.27°C, respectively) of temperature. The estimates are based on a logistic regression with date and week-NUTS2 fixed effects included and robust standard errors clustered at the region level. The horizontal lines show the 95% confidence intervals. N = 105,512.

**
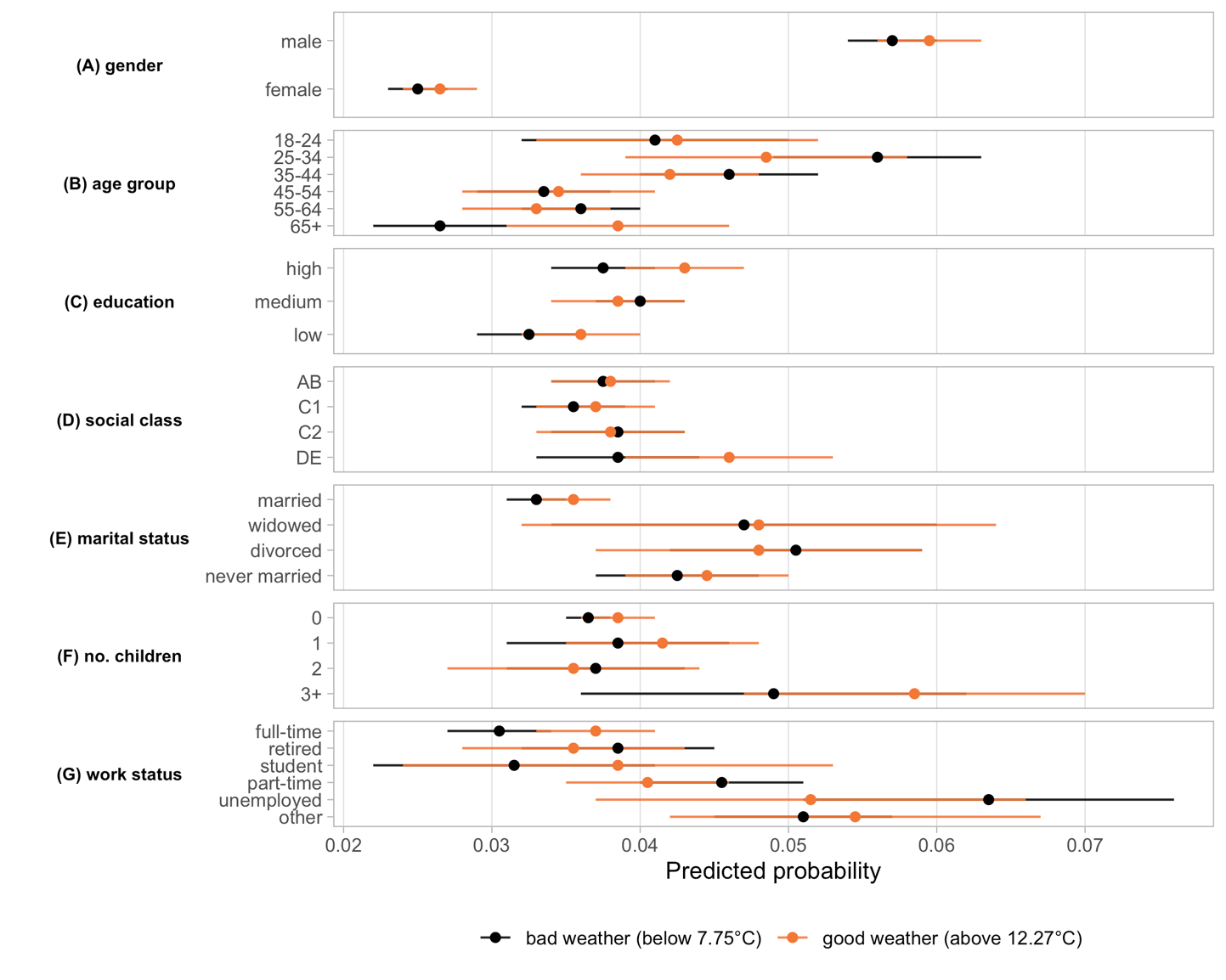
**

**SI. Fig. 9. Re-shuffling the original temperature variable leads to insignificant differences across all independent variables (placebo test).** The panels show the predicted probability of non-compliance when a given individual level characteristic being equal to 1 while all other covariates are held constant at their mean values. The black (orange) dots are estimates based on observations under bad (good) weather conditions, which are defined along the top and bottom quartiles (7.75°C and 12.27°C, respectively) of temperature. The estimates are based on a logistic regression with date and region fixed effects included and robust standard errors clustered at the region level. The horizontal lines show the 95% confidence intervals. N = 105,512.
